# Supplementary material for: Neuronal LXR Regulates Neuregulin 1 Expression and Sciatic Nerve-Associated Cell Signaling in Western Diet-fed Rodents
Source: Sci Rep. 2020 Apr 14;10:6396. doi: 10.1038/s41598-020-63357-1 (PMC7156713; doi:10.1038/s41598-020-63357-1)
Supplement: Supplementary file 1 — Supplementary information. [file 41598_2020_63357_MOESM1_ESM.docx]

**Neuronal LXR Regulates Neuregulin 1 Expression and Sciatic Nerve-Associated Cell Signaling in Western Diet-fed Rodents**

Chaitanya K. Gavini^1^, Raiza Bonomo^1^, Virginie Mansuy-Aubert^1*^

^1^Cell and Molecular Physiology, Stritch School of Medicine, Loyola University Chicago, Maywood, Illinois, USA 60153

^*^Corresponding author: Virginie Mansuy-Aubert; Cell and Molecular Physiology, Stritch School of Medicine, Loyola University Chicago, Maywood, Illinois, USA 60153

Phone: +1 (708) 216 - 3609; email: [vmansuyaubert@luc.edu](mailto:vmansuyaubert@luc.edu)

Supplementary data: RNA-seq data file, SN NC_vs_WD_Differential_Expression.xlsx
